# Charge to the Graduates in Medicine

**Published:** 1889-05

**Authors:** T. S. Powell

**Affiliations:** President of Board of Trustees


### Southern Medical Record.

A MONTHLY JOURNAL OF PRACTICAL MEDICINE.

☞ All communications must be addressed to R. C. WORD, Managing Editor.

" *Quicquid Præcipies Esto Brevis.*"

VOL. XIX ATLANTA, GA., MAY, 1889. NO. 5

### ORIGINAL AND SELECTED ARTICLES.

### CHARGE TO THE GRADUATES IN MEDICINE,

*At Southern Medical College Commencement, March, 2d, 1889.*

BY PROF. T. S. POWELL, M. D., PRESIDENT OF BOARD OF TRUSTEES.

MY YOUNG BRETHREN:

To night marks one of the most important eras in your life. It remains with you to determine whether its opening chapter of so much promise, and great possibilities shall prove the faithful horoscope of abundant fulfilment.

By the laws of the State of Georgia, you are now Doctors of Medicine.

This time-honored title is of much greater significance than we are wont to give it. To be a doctor of medicine—a true physician in its highest sense, is an attainment not lightly won, and is one of the most responsible and sacred obligations fallible man can take upon himself.

Are you prepared to invest the profession you have chosen with all the insignia of its true, exalted office? Have you well considered the purity of your motives in making this choice?—the stainless honor of the legitimate methods which must give the profession pre-eminence?—the tender humanity of its ministry?—the beautiful but inevitable self-sacrifice involved, and the motive power of indomitable perseverance which is necessary to win the crown that awaits one at the goal of a noble ambition?

The scope and possibilities of medical science are almost unlimited, and it must be admitted that the profession is not quite up to the progressive spirit of the age. As the people advance, we must also go forward. All other branches of science are now so fully enthused with a spirit of emulation, it seems a combined race among them to arrive at a greater and still greater degree of perfection.

The medical profession in extenuation of its tardy progress has said that its advancement was as great as the people were prepared to receive. But we must make a new departure, and prepare the minds of the people ourselves to accept and appreciate the spirit of modern progress in medicine.

It is true that ancient medical days were profoundly esteemed and venerated by the people, but with the superstition of the times, they wore about it a veil of mystery which the eye of modern progress has penetrated. Modern medicine does not seek to conceal its revelations from the laity, and they are at liberty to see that it is not a system of false fossilized ideas and expert jugglery for the patronage of a credulous public.

This is an intensely realistic age—almost too utilitarian in some things, but not in medicine. Years ago, common sense was rather at a discount, but now it takes a premium. True medical science is common sense in the full dress of natural laws, natural results, either as rewards or penalties, logical deductions and logical demonstrations. It has for its motto.

"The course of nature is the art of God."

If a physician finds that lemons in rheumatism and small pox; a strict diet of milk in Bright's disease; and oranges, tomatoes, and cranberries in torpidity of the liver are more efficacious than some time honored drugs in the pharmacopœia on scientific principles, by supplying that which is needed, and lessening that which is redundancy, we have not only acted in accord with nature, but in accord with the dictates of reason and of common sense, while the remedies prescribed, though simple and pleasant are not less potent and effectual agents.

It has been often said that nature has provided a remedy for every disease; and as she is always beneficent in her ministrations, why should the practitioner give to a sick man a nauseous compound drug, when a more pleasant one, one too, demanded upon scientific principles by the laws of health, may better accomplish the object in view.

The long, grave professional face in the sick-room, is now "out of style" as the ladies would say, and cannot deceive the acuteness of the intelligent patient with the impression of great occult knowledge behind the mask.

The profession you have chosen, my young brethren, in all its entirety, when rightfully estimated is one of such beneficent and beautiful ministry, and so replete with the marvelous foresight of an all-wise Creator there is nothing about it, under proper circumstances, that would justify deception or concealment.

On the contrary, if at the outset of your career you emulate the acme of professional knowledge, the patient and intelligent research of the wonderful realm of nature in all that pertains to the physical, moral and mental economy of man, and boldly make her hidden treasures your own, your revelations will be an unspeakable blessing to the world, and invest your name and your life with honor and veneration.

As I have said, the winning of your diplomas is but the opening chapter in the book of your professional life. There are many others to be lived and recorded before the last page is written, and it remains with you to determine what the end will be.

As there is a human and a divine side in all that pertains to the religion of Christ, so there is in the intelligent and conscientious study of medicine; and I have often thought when the life long student in the profession thus holds constant communion with nature, it would naturally lead the mind still higher up to nature's God, and therefore give to the character of the physician the refining and energizing stimulus which should distinguish that of every doctor of medicine.

Men sometimes express regret at having no legacy to leave their children, and no great deeds to their country's remembrance. But as John Winthrop says: "The noblest contribution which any man can make for the benefit of posterity is that of a good character." Lord Lyons asserts that a man is already of much consequence in the world, when it is known that we can implicitly depend upon him.

This knowledge can be given out only by the man's character, and the man himself must wield the chisel, and be the sculpter of that fair structure of adamantine solidity.

You may lose everything else on earth, but your good character will live through the cycles of time—it will live beyond the stars. It is more priceless than rubies, and, like wisdom, more to be desired than fine gold.

The true basis of this noble individuality is to always pursue the right, for the right's sake, no matter what the resutls may be; never to do wrong because it is politic, or because it may secure financial success, or ambitious position—never to do wrong because you cannot afford to do right.

Consistency is indeed a jewel, and gives strength and solidity to character, therefore, never advocate a wrong to serve a friend and then extol the right to accomplish some selfish, individual purpose. Even the dumb brutes of the forest have an amount of animal or physical courage, but moral courage is the sublimity of the truly brave spirit in man which fears nothing but God and the scorpion sting of a guilty conscience.

There is a vanity which desires the approbation of men whether it is or is not deserved; but that which inspires our efforts to be worthy of commendation, is a spur to the noble aspirations of him who deservedly wins the approval of his friends and the public. So there is a jealousy which tries to detract from the character and position of a brother because it happens you do not like him personally or professionally, and this is a baneful, ignoble spirit, while to be jealous of one's rights—to maintain them by commendable efforts and be worthy of their possession, is our privilege and imperative duty.

It is such principles as these that must reform what is unjust, unfraternal and illegitimate in the profession and exalt it to its true, noble, and dignified position.

As some one has said, the surest way to reform the world is for each one to reform himself. The high character of our fraternity must be built by individual efforts.

The interpretation of the word physician is emphatically that of a healer. What a desirable, beautiful mission that is, my young friends. The true doctor of medicine, in love with his profession—consecrated to its high office and sublime purposes, finds his chief joy, the scope of his noblest ambition in being an evangelist of compassion and healing to suffering man. In pursuance of this noble career, jealousies may persecute and seek to impede your advancement and lessen your usefulness; dissensions may divide you from those you have most loved and trusted, and you may be wounded even in the house of your friends, but let none of these things deter you from keeping your feet resolutely moving on in the path of duty, illumined by the ever shining star of truth.

Fix your eyes upon its undying light—truth in medicine, truth in principle, character, and in all things; and make it your noble purpose to elevate your profession, morally and intellectually to the honored, exalted place it should have, and can have in the eyes and the affections of the people.

Though the aggregate members of the medical ranks may not com bine their forces to reform the abuses in the profession, and war against everything that would lower its standard, you can add your own individual efforts to the quota of fearless spirits which strive not alone for their own exaltation, but for that of their loved profession.

You will have your reward, even in this world, the good and true men will sustain you—and then when all earthly honors dissolve from view like the mists of a dream, may infinite love and justice give you a crown which fades not away in that better land beyond the stars.

